# Commentary: The Turnaway Study: A case of self-correction in science upended by political motivation and unvetted findings

**DOI:** 10.3389/fpsyg.2022.1003116

**Published:** 2022-12-02

**Authors:** Antonia Biggs, Diana Greene Foster, Heather Gould, Katrina Kimport, Lauren Ralph, Sarah Roberts, Corinne Rocca, Gretchen Sisson, Ushma Upadhyay, Katie Woodruff

**Affiliations:** Department of Obstetrics, Gynecology and Reproductive Sciences, University of California, San Francisco, San Francisco, CA, United States

**Keywords:** abortion, longitudinal study design, study recruitment, mental health, research

## Introduction

The Turnaway Study—a longitudinal study of people recruited from 30 abortion care facilities across the United States from 2008 to 2016—has resulted in over 50 publications rigorously detailing the consequences of receiving vs. being denied an abortion on women's health and socioeconomic wellbeing^1^. Findings have been published in top peer-reviewed medical, public health and sociology journals. In this paper we respond to the methodological critiques raised by Priscilla Coleman (see Table 1) (Coleman, 2022).

**Table 1:** Coleman's critiques of Turnaway Study methodology.

1. She claims that the study population represents a tiny fraction of the total abortions sought at the recruitment clinics
2. She raises concern about the potential for selection bias whereby those with the potential for mental health problems may have been excluded
3. She asserts that the number of people participating in the study has been overstated
4. She claims that many of the outcome measures were assessed too simplistically
5. She asserts that people seeking abortions at different gestations were inappropriately grouped together
6. She suggests that it would be appropriate to examine outcomes separately for people who had previously had an abortion vs. those who had one after giving birth
7. She criticizes the lack of a comparison group of women who have chosen not to seek an abortion

## Addressing the criticisms

Our goal was not to recruit all people seeking abortion over the study period. We specifically recruited women just above and just below the gestational limit, rather than all women seeking abortion to ensure unbiased estimates of the effects of abortion on women's lives. By comparing the outcomes of similar groups of women who, almost at random, received or were denied their wanted abortion, we were able to isolate the effects of abortion on people's wellbeing.

For a study to be generalizable, the characteristics of the sample need to reflect the target population of people seeking abortions and any selection biases need to be minimized. Our sample closely reflects the characteristics of those seeking abortion nationally. We examined outcomes for women recruited from high vs. low participation rate sites and did not find differences.

We asked clinics to approach every woman who was denied an abortion due to being over the gestational limit. For the other two groups, for which eligibility was more common, clinics were permitted to select times to recruit so long as they approached every eligible person while they were doing recruitment. This flexibility around recruitment for the two abortion groups explains the lower approach rates for those who received abortions. As we have previously reported, clinics were not deciding who to approach based on any characteristics of the abortion patient other than eligibility for one of the three study groups (Dobkin et al., 2014).

It is inappropriate to characterize a study's sample size based solely on the number of people who complete the final interview. With longitudinal studies, there is inevitable loss to follow up. Loss to follow up is a concern when those lost are systematically different from those who remained in the study; in Turnaway we have evidence that loss to follow up was not different by baseline physical health, mental health, or economic wellbeing. The Turnaway Study collected data for over 1,000 people. Some did not do the phone interviews and were included in the credit study and death record search. Nine hundred fifty-six completed at least one phone interview. We use all 7,851 interviews in our analyses which allows us to show that that there was not differential loss to follow up based on attitude toward abortion or key outcomes. The mixed effects regression models we employ in our analyses account for missing observations, protecting against bias owing to loss to follow up that is predictable from previously measured covariates.

Although Coleman criticizes the Turnaway Study's participation and attrition rates, these rates are well within the expected range for a five-year study, and similar to other prospective studies of this type (Bao et al., 2016). Our rate of attrition of about five percent from wave to wave represents excellent participant retention compared to other research in the field and is a study strength. Coleman draws attention to a small difference we found in who was lost at 3 years (Rocca et al., 2015). Yet we have also reported that by 5 years, there was no longer a difference in retention by baseline emotions or any other related outcome: decision rightness, decision difficulty, or perceived abortion stigma (Rocca et al., 2020).

All our mental health measures are widely accepted and have been validated in the United States and elsewhere: the Brief-Symptom Inventory (BSI) (Boulet, 1991), the Patient Health Questionnaire-9 (Kroenke et al., 2001), the Primary Care PTSD Screen (PC-PTSD) (Prins et al., 2003), and the Sheehan Suicidality Tracking Scale (Coric et al., 2009). Our only one-item measure, the self-esteem scale, is reliable and valid and is recommended as a practical alternative to the ten-item Rosenberg Self-Esteem scale (Robins et al., 2001).

The criticism that we have lumped participants who have early and later abortions together is false. In nearly all the papers, we present the data by the original study groups—those over the gestational limit who were denied an abortion, those just under the limit who receive an abortion, and those in the first trimester. But the underlying assumption of the critique—that women who seek later abortions have different psychological responses than people who seek early abortions–is not supported by Turnaway Study findings. When explicitly comparing reason for abortion, emotional reactions and decision rightness among people seeking near-limit vs. first-trimester abortions, we find few differences (Biggs et al., 2013; Rocca et al., 2015). Instead, we find that people seeking abortion later in pregnancy do so primarily because they discovered they were pregnant later and because they experienced a series of logistical and financial barriers to getting an abortion (Upadhyay et al., 2014; Foster et al., 2021).

The Turnaway Study captures experiences of people who were seeking abortion services and therefore would be affected by abortion bans. Any selection of subsets of people who never had a prior abortion or never go on to have another unwanted pregnancy would create biases that would result in the sample no longer reflecting all those who sought an abortion. To understand the effect of access to abortion on people's lives, there is no reason to include a sample who choose to carry unintended pregnancies to term. These people are not affected by the availability of abortion services.

## Discussion

The most important contribution of the Turnaway study is the quasi-randomized design: that women were similar to each other before one group got an abortion and the other was denied (and, based on credit reports, were similar for 3 years before the study started). The ways in which their lives diverged are therefore directly attributable to whether they got their abortion or not. We find large negative economic (Foster et al., 2018; Miller et al., 2020) and health (Gerdts et al., 2016; Ralph et al., 2019) consequences for people who are denied wanted abortions, an experience that is going to be more common now that *Roe v Wade* has been overturned and many states have banned abortion.

## Author contributions

DF drafted the commentary with input from her co-authors. All authors contributed to the article and approved the submitted version.

## Conflict of interest

The authors declare that the research was conducted in the absence of any commercial or financial relationships that could be construed as a potential conflict of interest.

## Publisher's note

All claims expressed in this article are solely those of the authors and do not necessarily represent those of their affiliated organizations, or those of the publisher, the editors and the reviewers. Any product that may be evaluated in this article, or claim that may be made by its manufacturer, is not guaranteed or endorsed by the publisher.

## Footnotes

^1^https://www.ansirh.org/sites/default/files/publications/files/turnawaystudyannotatedbibliography.pdf (accessed July 26, 2022).
